# Gastric perforation following cytoreductive surgery and perioperative intraperitoneal chemotherapy: a case series of six

**DOI:** 10.1186/s12957-017-1114-7

**Published:** 2017-02-10

**Authors:** Lee S. Kyang, Nayef A. Alzahrani, Jing Zhao, David L. Morris

**Affiliations:** 1 Department of Surgery, St. George Hospital, University of New South Wales, Sydney, New South Wales Australia; 2 Imam Muhammad ibn Saud Islamic University College of Medicine, Riyadh, Saudi Arabia

**Keywords:** Peritonectomy, Nasogastric tube, Suction, Heated intraoperative intraperitoneal chemotherapy, HIPEC, Appendiceal cancer, Gastric perforation, Stomach

## Abstract

**Background:**

Incidence of gastric perforation following cytoreductive surgery (CRS) and perioperative intraperitoneal chemotherapy (PIC) is not widely reported.

**Methods:**

Suitable patients were identified from our database of 1028 procedures. Relevant information was then gathered via medical records and operation reports for these patients.

**Results:**

Six patients suffered early postoperative gastric perforation following the procedure (0.58%), all of whom received heated intraoperative intraperitoneal chemotherapy (HIPEC). Surgical exploration revealed protrusion of nasogastric (NG) tube through stomach wall defects which were either located at or near the greater curvature of stomach. These patients were managed successfully with operation, and no mortality was recorded.

**Conclusions:**

Gastric perforation following CRS and PIC is most likely the result of a multifactorial process. To reduce the risk of such complication, avoiding nasogastric suction in these patients may prove helpful. Any suspected perforated viscus must be addressed promptly to avoid unwanted morbidity and mortality from the procedure. To our knowledge, conservative management has not been documented to work in this subgroup and surgery remains the mainstay of treatment.

## Background

Historically, the prognosis of peritoneal dissemination of neoplasms (primary or metastatic), also known as peritoneal carcinomatosis (PC), was poor. Over the past few decades, the introduction of cytoreductive surgery (CRS) and perioperative intraperitoneal chemotherapy (PIC) has significantly altered the treatment landscape for PC and, owing to its promising survival benefits [1–3], the combination treatment has gained popularity. Essentially, there are two major components to the procedure: first, CRS involves removal of macroscopic tumour off the peritoneum and/or visceral organs; then, intraoperative chemotherapy (HIPEC) or postoperative chemotherapy (EPIC) allows a high concentration of cytotoxic drug to destroy microscopic residual disease [4].

Despite well-documented survival results, perception towards the therapeutical approach remains sceptical due partly to its high toxicity [5]. However, clinical experience has allowed surgeons to improve the outcomes. A study at St. George Hospital in Sydney, which prospectively studied 140 patients who underwent CRS and PIC, demonstrated a significant reduction in morbidity from 30 to 10% and mortality from 7 to 1% when the former 70 patients were compared to latter 70 patients [6]. This data suggests a learning curve effect associated with the procedure. We have now done 1000 procedures and have a mortality of approximately 1% in the last 4 years.

The high morbidity and mortality rates of such intervention can be largely attributed to the surgery and/or chemotherapy [7]. Multiple body systems can be impacted and gastrointestinal complications are the most prevalent including abscess (0–37%), fistula (0–23%), ileus (0–86%), anastomotic leak (0–9%) and bowel perforation (0–10%) [4]. To our knowledge, there are, however, only a few reports of gastric perforation in these patients. Here we outline and evaluate our experience with six patients complicated by gastric perforation following CRS and PIC.

## Methods

We retrospectively explore a prospectively maintained database of 843 patients, amounting to 1028 procedures, who underwent CRS and perioperative chemotherapy for intraperitoneal dissemination of primary cancers at St. George Hospital (Sydney) from 1996 to June 2016 to identify patients with postoperative gastric perforation. Medical records and operation reports for the identified patients were then reviewed to gather relevant information for this case series. All our patients are preoperatively consented to have information stored in our database for research purposes (by South Eastern Sydney Local Health District Human Research Ethics Committee).

## Results

All the procedures were done by one surgical team. These patients were reviewed by a multidisciplinary team of surgical oncologists, medical oncologists, anaesthetist, radiologists, nurses and allied health members. So far, six incidents of postoperative gastric perforations were reported following CRS and perioperative chemotherapy. The patients consisted of two men and four women with a mean age of 50.7 (41–62 years old), representing an incidence of 0.58% (6/1028) from our database. The initial CRS were performed to remove pseudomyxoma peritoneii (appendiceal neoplasm) in three patients, peritoneal mesothelioma in one patient and ovarian cancer in the remaining two. During the surgeries, variable procedures were done (Table 1), determined by the distribution, volume and invasion of PC. Mean peritoneal cancer index (PCI), as defined by Jacquet and Sugarbaker [8], was 27.5 in these patients. A mean time of 9.6 h was required to operate on these patients.

**Table 1:** Characteristics of patients with gastric perforation following CRS and PIC at a tertiary referral centre (St. George Hospital) in Sydney

Patient number; age; gender	Diagnosis	Procedures performed	PCI^a^	HIPEC^b^ (chemotherapy)	EPIC^c^	Length of surgery (hours)
1; 62; M	Pseudomyxoma peritoneii (redo)	Peritonectomy, small bowel resection	17	Yes (MMC^d^)	No	8.5
2; 58; F	Ovarian cancer	Peritonectomy, splenectomy, cholecystectomy, partial gastrectomy with Roux-En-Y anastomosis, small bowel resection, bilateral diaphragm strip	32	Yes (CDDP^e^)	No	10.5
3; 41; F	Pseudomyxoma peritoneii	Peritonectomy, splenectomy, cholecystectomy, omentectomy, right hemicolectomy	14	Yes (MMC)	Yes	10.0
4; 51; F	Peritoneal mesothelioma	Peritonectomy, bilateral diaphgram stripping, splenectomy, right hemicolectomy, cholecystectomy, segment II liver resection, pelvic stripping, omentectomy	33	Yes (CDDP)	No	9.0
5; 44; M	Pseudomyxoma peritoneii	Peritonectomy, bilateral diaphragmatic stripping, splenectomy, pancreas stripping, liver surface stripping, cholecystectomy, Billroth I gastrectomy, right hemicolectomy, anterior resection	39	Yes (OX^f^)	No	12.0
6; 48; F	Ovarian cancer	Peritonectomy, oophorectomy, salpingectomy, salpingooophorectomy, removal of ligaments (ovarian, paraovarian, fimbrial or broad ligaments), hysterectomy with rectum, bilateral diaphragm stripping, splenectomy, partial gastrectomy, left hepatectomy and creation of colostoma	30	Yes (OX)	No	7.8

^a^Peritoneal cancer index

^b^Hyperthermic intraperitoneal chemotherapy

^c^Early postoperative intraperitoneal chemotherapy

^d^Mitomycin C

^e^Cisplatin

^f^Oxaliplatin

Hyperthermic intraoperative intraperitoneal chemotherapy (HIPEC) was introduced in all six patients. Hyperthermic mitomycin C was given to patients 1 and 3, hyperthermic cisplatin was given to patients 2 and 4 and hyperthermic oxaliplatin was used in patients 5 and 6. In contrast, only one patient received early postoperative intraperitoneal 5-fluorouracil (patient 3) for a total of 5 days. Following the procedure, proton pump inhibitor (PPI) was prescribed for these patients till they are mobilised or discharged from hospital.

Diagnosis of gastric perforation was confirmed by direct visualisation of stomach wall defect. It took us a mean time of 6 days postoperatively to diagnose possible perforated viscus in these patients based on signs of peritonitis, fluid content in drain and CT abdomen (Table 2). These patients were brought to theatre for exploration and closure of perforation. We tried conservative management on one patient (patient 3), however, with no clinical improvement. Ultimately, she underwent surgical exploration to fix the perforation.

**Table 2:** Operative and post-operative characteristics of the same set of patients

Patient number; age; gender	Time from initial CRS to perforation diagnosis (days)	Indications of perforated viscus	Surgery or conservative	Location of stomach perforation	How was it fixed?	Length of hospital stay (days)
1; 62; M	2	Brown fluid in drain	Surgery	5 mm adjacent to liver edge	Oversewn with vicryl	24
2; 58; F	9	Ongoing peritonism with brownish discharge from abdominal wound despite unremarkable CT	Surgery	Above gastroenterostomy	Oversewn then with diaphragm patch	55
3; 41; F	7	Peritonism, green billous fluid in drain	Conservative then surgery	Stomach body	Oversewn with vicryl	44
4; 51; F	10	Peritonism, CT abdomen, green billous fluid in drain	Surgery	3 mm, greater curvature of stomach	Oversewn with menseteric fat	26
5; 44; M	6	Peritonism, CT abdomen	Surgery	5 mm, greater curvature of proximal stomach	Oversewn with vicryl and plication	34
6; 48; F	3	Green billous fluid in drain	Surgery	5 mm, posterior gastric wall 1 cm away from greater curvature	Oversewn with vicryl and plication	35

**Fig. 1:**
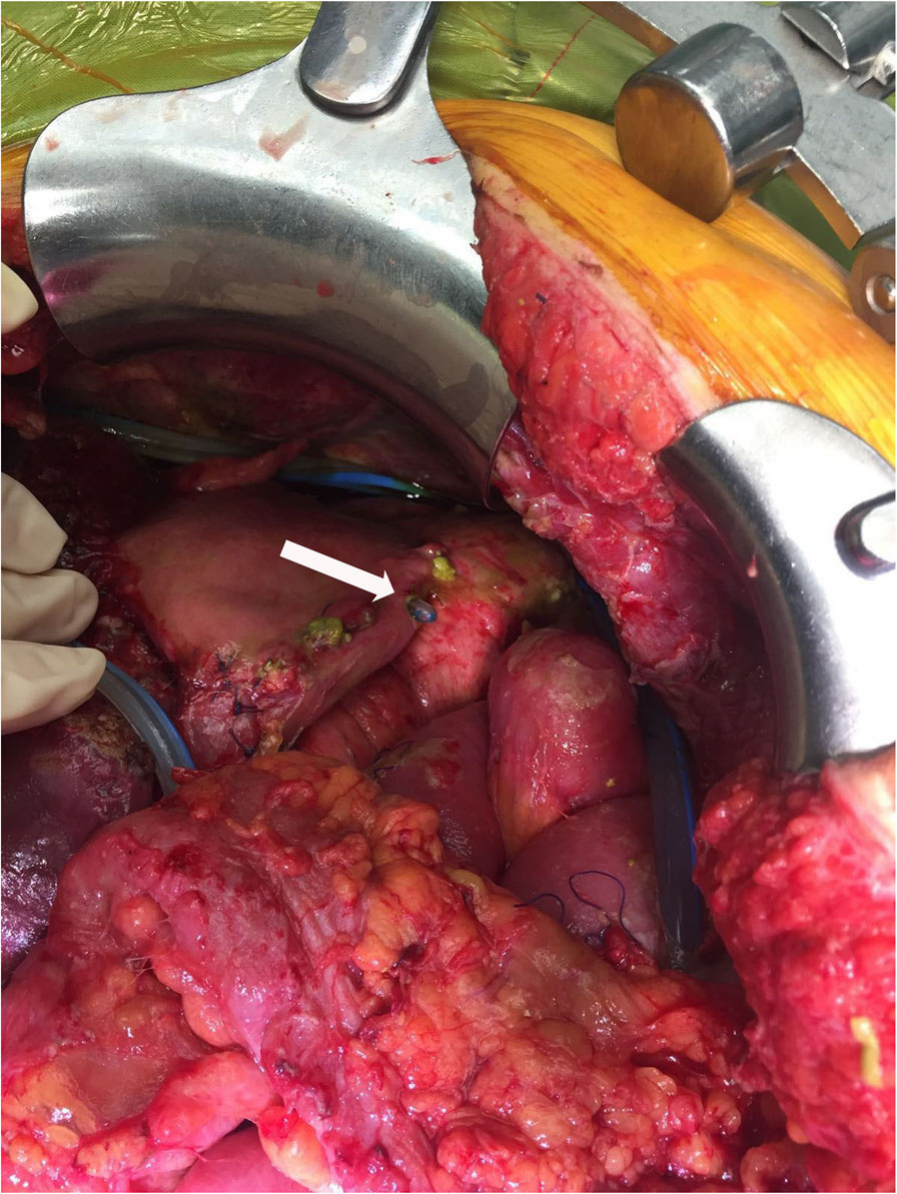
Postoperative nasogastric tube perforation (*arrow*), through the greater curvature of stomach, leading to bile peritonitis in patients who underwent CRS and HIPEC

All gastric perforations were either located at or near the greater curvature of stomach (Fig. 1). The sizes of the defects were no more than 0.5 cm in diameter and NG tube was seen protruding through the stomach, causing bile peritonitis in all cases. The defects were repaired by suturing the perforation on two layers. The outcome for these patients was positive with no mortality. The mean length of hospital stay for these patients was 36 days. Patient 2 had the longest in-hospital admission (55 days) due to concurrent complications of gastrointestinal bleeding and intra-abdominal abscess.

## Discussion

We retrospectively explored the incidence of gastric perforation following CRS and PIC from our prospective database. In our institution, out of the 1028 procedures performed in the last two decades, six patients (0.58%) had postoperative recovery complicated by gastric perforation. These patients were taken back to theatre for emergency repair of perforated viscus. All gastric perforations were either at or near the greater curvature of stomach. Fortunately, all of them achieved complete resolution and the mean length of hospital stay for these patients was 36 days. In contrast to bowel perforation, postoperative gastric perforation associated with CRS and PIC is a relatively rare surgical complication. To our knowledge, this has not been widely reported in the literature. So far, only eight patients were described to have gastric perforation following CRS and PIC (Table 3).

**Table 3:** Available literature documenting incidence of gastric perforation following CRS and PIC

Reference	Study type	Aim	Number	Early or delayed complication	Origin	HIPEC	Mortality from complication	Management	Suggested possible mechanism
Kusamura et. al. [12]	Retrospective observational study	To analyse morbidity and mortality of CRS and intraoperative hyperthermic infusion in treatment of peritoneal malignancies	1/209 procedures	UC^a^	UC	Yes (UC)	No	Surgical	• Partial thickness mechanical and/or thermal damage to visceral surface (aggravated by heated chemotherapy) • Focal heat injury at tip of inflow catheter • Mechanical trauma due to suctioning effect of outflow catheter • Post-operative shrinking of infiltrating metastatic nodules on visceral wall from antiblastic effect of heated chemotherapy
Ceelen et. al. [20]	Prospective study	To analyse safety and efficacy of HIPEC using high dose oxaliplatin in CRS	1/52 patients	UC	UC	Yes (OX)	No	Surgical	Thermal damage during omentectomy using ultrasonic shears
Zappa et. al. [9]	Retrospective observational study	To explore the cause and management of gastric perforation following CRS and HIPEC	4/1251 patients	Early	• 3 appendiceal cancer • 1 ovarian cancer	Yes (1—MMC; 2—MMC + DOX^c^; 1—CDDP	No	Surgical	• Local seromuscular trauma to the greater curvature as a result of traction on the ligated blood vessel and vascular compromise • Damage of stomach wall from direct effects of chemotherapy, which is further amplified by poor perfusion • Nasogastric trauma secondary to suction during postoperative phase
Bhagwandin et. al. [13]	Retrospective observational study	To analyse incidence of delayed major complications of CRS and HIPEC post-discharge	1/140 procedures	Delayed	Mesothelioma	Yes (cisplatin, DOX)	No	Endoscopic clipping	NR^b^
Munoz-Casares et. al. [14]	Retrospective observational study	To analyse long-term outcomes of CRS plus HIPEC	1/218 patients	Delayed	Ovarian cancer	Yes (paclitaxel)	No	Surgical	NR
Martin et. al. [15]	Retrospective observational study	To identify variables associated with readmission rates following CRS and HIPEC	NR	Delayed	UC	Yes (NR)	Yes (1 sepsis)	NR	NR

^a^Unclear

^b^Not reported

^c^Doxorubicin

The location of stomach defect at or near the greater curvature indicates possible pathology at that area. All our patients, except patient 1, had either omentectomy, splenectomy or both done during the procedure. During the procedure, ligations of right and left gastroepiploic vessels (greater omentectomy) and splenic vessels (splenectomy) reduce perfusion of greater curvature, resulting in seromuscular trauma at site of ligation due to traction [9]. We also strongly believe that the injury may be associated with nasogastric tube based on the visualisation of tube protrusion through the stomach defects during exploratory laparotomy. Perhaps it was related to pressure ischemia exerted on stomach mucosa by nasogastric tube suction and by relatively rigid nasogastric tubes [10]. Other proposed mechanisms include direct effect of intraperitoneal chemotherapy [11] and thermal injury caused by inflow and outflow catheters during infusion of HIPEC [12]. All these factors, ultimately, lead to friability of stomach wall making it susceptible to perforation.

Postoperative monitoring for any indication of perforated viscus is paramount. Signs of peritonitis, fluid content in intraperitoneal drain and CT abdomen can be useful to guide our suspicion. We would like to highlight, though, that false negative is still possible on CT abdomen and complete clinical presentation must be taken into account to establish index of suspicion, in addition to imaging. For instance, in patient 2, we decided to perform surgical exploration due to continuous sepsis (temperature >40 °C) and feculent discharge from abdominal wound despite unremarkable CT finding. In contrast, the delayed diagnosis in patient 4 was rather unfortunate. Spiked temperature postoperatively was initially thought to result from hospital-acquired pneumonia (*Pseudomonas*-positive on sputum culture). Because of unresolved fever despite being on optimal antibiotic therapy, an abdominal CT imaging was performed, which showed a defect in lateral wall of stomach, and an urgent surgical intervention was conducted. Surprisingly, the patient’s recovery was unremarkable and had a relatively short postsurgical stay.

All six gastric perforations were diagnosed in the early postoperative phase (mean of 6 days). However, there were at least three incidents of gastric perforation described in the literature that manifested as a long-term complication of CRS and PIC [13–15]. The documented events occurred following discharge of patients from hospital. At our institution, all patients are monitored monthly for the first 3 months after discharge and six monthly thereafter, during which clinical examination and review of pertinent tumour markers are conducted. Thus far, we have not encountered a single case of gastric perforation throughout follow-up period following discharge for patients with CRS and PIC. However, we acknowledge and concur with Bhagwandi et al. [13] that possible occurrence of such life-threatening complication while patients are no longer monitored by the treating surgeon could lead to unnecessarily high morbidity and mortality. Therefore, follow-up schedule should be well established in each peritonectomy institution to track patients’ postoperative progress.

Nonoperative management of gastric perforation was proven to be viable by Crofts et al. [16] in managing patients with perforated gastric ulcer, dating back to 1989. It has been studied extensively in patients with perforated peptic ulcers, and the results have been promising when compared to operative management [17, 18]. This alternative can be executed by keeping the stomach empty through a strict nil-by-mouth regime and nasogastric aspiration, in adjunct with close monitoring of patient’s clinical status and administration of antibiotic and PPI [19]. We tried conservative management on one patient (patient 3). A lack of clinical improvement prompted immediate surgical intervention. Similarly, as outlined in Table 3, none of the patients were managed conservatively in the literature. As the nonoperative management hinges on allowing the perforated site to heal and seal by itself [19], the impact of HIPEC on this is currently not clear. Regardless of the mechanism, CRS and HIPEC are suggested to result in a weaker stomach wall, at least in the immediate postoperative phase. It would be sensible to refrain exerting more pressure on the stomach mucosa. Therefore, we propose to avoid suction on NG tubes following the procedure.

## Conclusions

Gastric perforation is a rare surgical complication following CRS and PIC, and it is most likely the result of a multifactorial process. To reduce the risk of such complication, avoiding nasogastric suction in these patients may prove helpful. Nevertheless, any suspected perforated viscus must be addressed promptly to avoid unwanted morbidity and mortality from the procedure. To our knowledge, conservative management has not been documented to work in this subgroup and surgery remains the mainstay of treatment.

## Abbreviations

CRS: Cytoreductive surgery
HIPEC: Heated intraoperative intraperitoneal chemotherapy
PC: Peritoneal carcinomatosis
PCI: Mean peritoneal cancer index
PPI: Proton pump inhibitor
